# Defined α-synuclein prion-like molecular assemblies spreading in cell culture

**DOI:** 10.1186/1471-2202-15-69

**Published:** 2014-06-04

**Authors:** Suzana Aulić, Tran Thanh Nhat Le, Fabio Moda, Saïda Abounit, Stefania Corvaglia, Loredana Casalis, Stefano Gustincich, Chiara Zurzolo, Fabrizio Tagliavini, Giuseppe Legname

**Affiliations:** 1 Laboratory of Prion Biology, Department of Neuroscience, Scuola Internazionale Superiore di Studi Avanzati (SISSA), via Bonomea 265, 34136 Trieste, Italy; 2 Division of Neuropathology and Neurology 5, IRCCS Foundation Carlo Besta Neurological Institute, Via Celoria 11, 20133 Milan, Italy; 3 Trafic Membranaire et Pathogenèse, Biologie des Interactions Cellulaires, Institut Pasteur, 25 rue du Docteur Roux, 75724 Paris CEDEX 15, France; 4 Elettra-Sincrotrone Trieste S.C.p.A., Area Science Park, 34149 Basovizza, Trieste, Italy; 5 Department of Neuroscience, International School for Advanced Studies (SISSA), Trieste, Italy

**Keywords:** α-Synuclein, Protein aggregation, Seeding, Prion

## Abstract

**Background:**

α-Synuclein (α-syn) plays a central role in the pathogenesis of synucleinopathies, a group of neurodegenerative disorders that includes Parkinson disease, dementia with Lewy bodies and multiple system atrophy. Several findings from cell culture and mouse experiments suggest intercellular α-syn transfer.

**Results:**

Through a methodology used to obtain synthetic mammalian prions, we tested whether recombinant human α-syn amyloids can promote prion-like accumulation in neuronal cell lines *in vitro.* A single exposure to amyloid fibrils of human α-syn was sufficient to induce aggregation of endogenous α-syn in human neuroblastoma SH-SY5Y cells. Remarkably, endogenous wild-type α-syn was sufficient for the formation of these aggregates, and overexpression of the protein was not required.

**Conclusions:**

Our results provide compelling evidence that endogenous α-syn can accumulate in cell culture after a single exposure to exogenous α-syn short amyloid fibrils. Importantly, using α-syn short amyloid fibrils as seed, endogenous α-syn aggregates and accumulates over several passages in cell culture, providing an excellent tool for potential therapeutic screening of pathogenic α-syn aggregates.

## Background

α-Synuclein (α-syn) is a key player in the pathogenesis of a group of neurodegenerative diseases defined as synucleinopathies, including Parkinson disease (PD), dementia with Lewy bodies (DLB), and multiple system atrophy (MSA). The discovery that aggregated α-syn is the major component of Lewy bodies (LBs) [1] indicates a role of α-syn in PD. Neurons containing LBs are the hallmark of PD and DLB, whereas in MSA α-syn is deposited in oligodendrocytes referred to as glial cytoplasmic inclusions (GCIs) [2-4]. In its aggregated form, α-syn is enriched in β-sheet structure, orderly organized into oligomers or amyloid fibrils [5]. As such, many amyloid proteins are characteristic of specific neurodegenerative disorders, for instance Alzheimer disease (AD) and prion diseases. For several decades it has been speculated that the key to understanding age-related neurodegenerative disorders may be found in the unusual biology of prion diseases. This hypothesis is being increasingly supported by experimental evidence. The self-propagation of amyloid-β (Aβ) aggregates is also known from *in vitro* studies [6] and inoculation experiments [7]. However, only recent experiments using genetically modified rodents have established that Aβ can be induced to deposit in brain by a prion-like mechanism. Intracerebral injection of Aβ-rich brain extracts from AD patients or from aged APP-transgenic mice stimulated the premature formation of plaques in these models [8,9]. Aβ lesions in APP transgenic mice are also inducible by injections of pure, synthetic human Aβ fibrils, although synthetic seeds are less powerful than aggregates formed within the living brain. Like prions, Aβ seeds vary in size from small, soluble, protease-sensitive aggregates to large, insoluble, protease-resistant fibrils [10].

Accumulating experimental data indicate that the seeding principle also applies to other pathogenic proteins that form amyloid-like inclusions within the cells. This is also the case of α-syn which, in its misfolded state, forms assemblies known as LBs [11]. In PD, α-syn aggregates arise first in the brainstem and then spread to telencephalic structures [12]—a dynamics indicative of prion-like spread of protein aggregation. Numerous studies have shown cell-to-cell transmission of soluble or aggregated α-syn, both in cultured cells and in mouse brains, resulting in α-syn aggregation and neuronal dysfunction in the recipient cells [6,13-15]. Importantly, injections of synthetic (human or mouse) α-syn fibrils also induce the recruitment of endogenous soluble α-syn protein to form LB-like pathology and neuronal degeneration in primary cell culture [16] and in non-transgenic (wild type) host mice [17,18]. Lastly, Melki *et al.* have shown that two different α-syn polymorphs (fibrils and ribbons) exhibit marked differences in their propensity to penetrate the cells, as well as in their toxicity and seeding aggregation in cells, suggesting the existence of different α-syn strains [19].

On the basis of these findings, we considered the possibility that recombinant human α-syn could acquire prion-like properties, once it is converted into a β-sheet-rich structure, and determine the fate of endogenous α-syn in human immortalized cell lines.

## Results

### Aggregation properties and structural characterization of recombinant α-syn assemblies

Evidence in literature shows that, under different experimental conditions, the formation of LB-like inclusions can be induced in cultured cells overexpressing human α-syn [20]. To test whether the same process may be induced in the non-transfected human SH-SY5Y cell line we applied different α-syn fibril assemblies to the cell culture.

**Figure 1:**
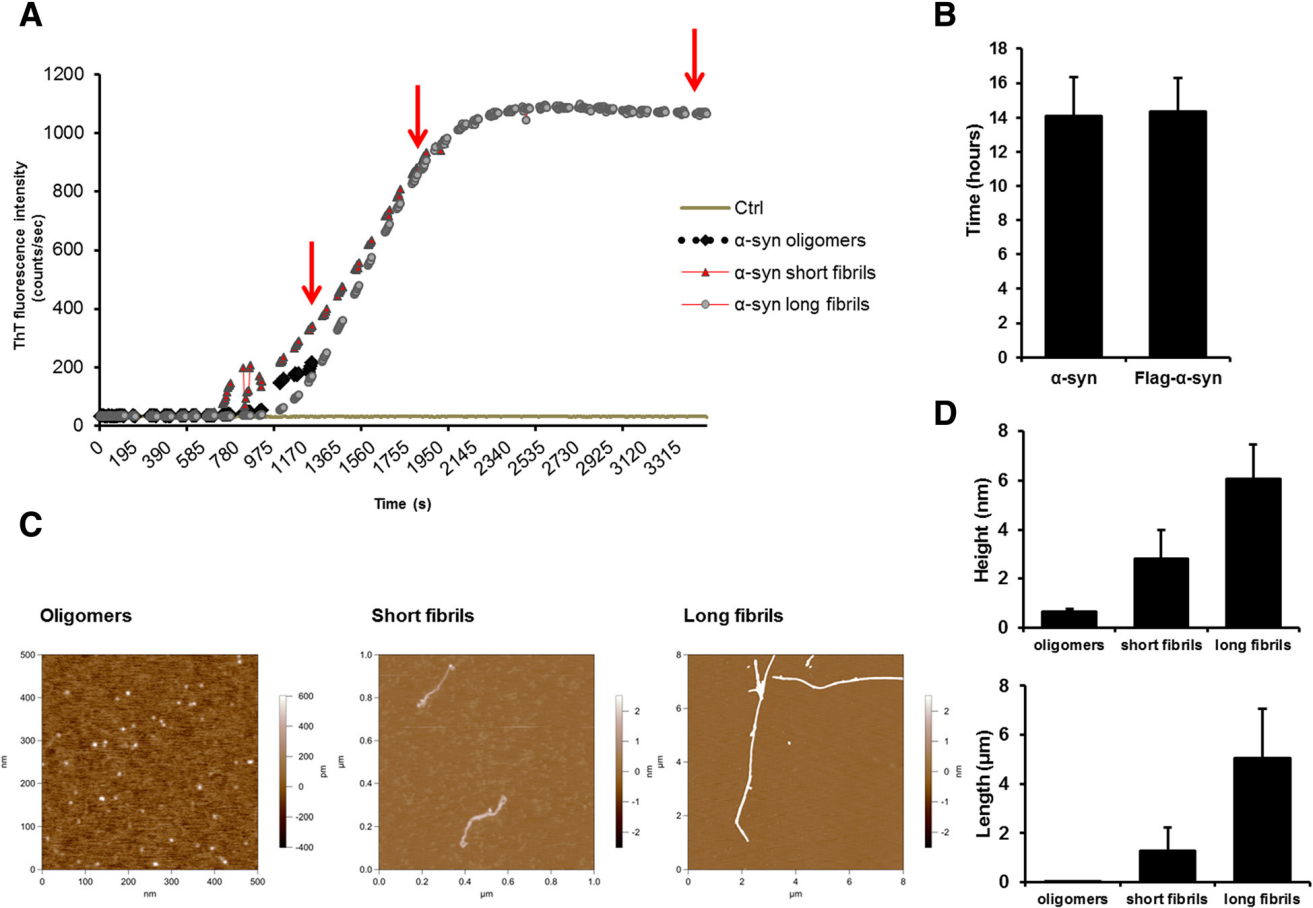
***In vitro *conversion and AFM characterization of three different recombinant α-syn assemblies.** Kinetics for the formation of β-sheet-rich assemblies: human α-syn oligomer (dotted black line), human α-syn short fibrils (red triangle line) and human α-syn long fibrils (gray dotted line), and control (gray solid line). **(A)** Red arrows indicate the collection time of the aggregates. **(B)** The lag phase, in hours, of all β-sheet structure preparations was measured using Thioflavin T assay. The lag phase distribution of α-syn and FLAG-α-syn amyloid preparations showed no difference (P > 0.5), indicating that the presence of FLAG-tag did not affect fibril formation. **(C)** AFM imaging analysis was performed at the end of the fibrillization reactions. AFM scan topographical images of α-syn deposited on mica surface. **(D)** The size of particles was measured: the typical height of α-syn oligomers was 0.65 ± 0.11 nm, the height of short and log fibrils was 2.79 ± 1.20 nm, and 6.08 ± 1.38 nm, respectively. The values are the average calculated on 20 fibrils. The error is the standard deviation.

First, we expressed in bacteria and then purified recombinant human α-syn protein (Additional file 1: Figure S1) either as wild-type sequence or tagged with a FLAG epitope, and established a protocol to induce structured amyloid assemblies as previously shown for the production of mammalian synthetic prions [21] (Figure 1A). Because various intermediate forms of α-syn develop during the process of fibril formation, we decided to collect β-sheet-rich structures at different time points during the fibrillization assay: (i) at the early inflection of the sigmoid curve; (ii) at the middle portion of the curve; and (iii) at plateau (collection times are highlighted by red arrows in Figure 1A).

We reasoned that these diverse β-sheet-rich assemblies might differ in quaternary structure. As predicted, atomic force microscopy measurements showed diverse assemblies: oligomers, and short and long amyloid fibrils (Figure 1C). From the statistical analysis of the three different preparations (Figure 1D) we identified three different assemblies enriched in: (i) oligomeric structure, indicating spherical, ring-like characteristics; (ii) short fibrils, the protofibril-like structure, and (iii) long fibrils (Additional file 2: Table S1 lists all height and length values observed).

### Toxicity of α-syn aggregates in cells

**Figure 2:**
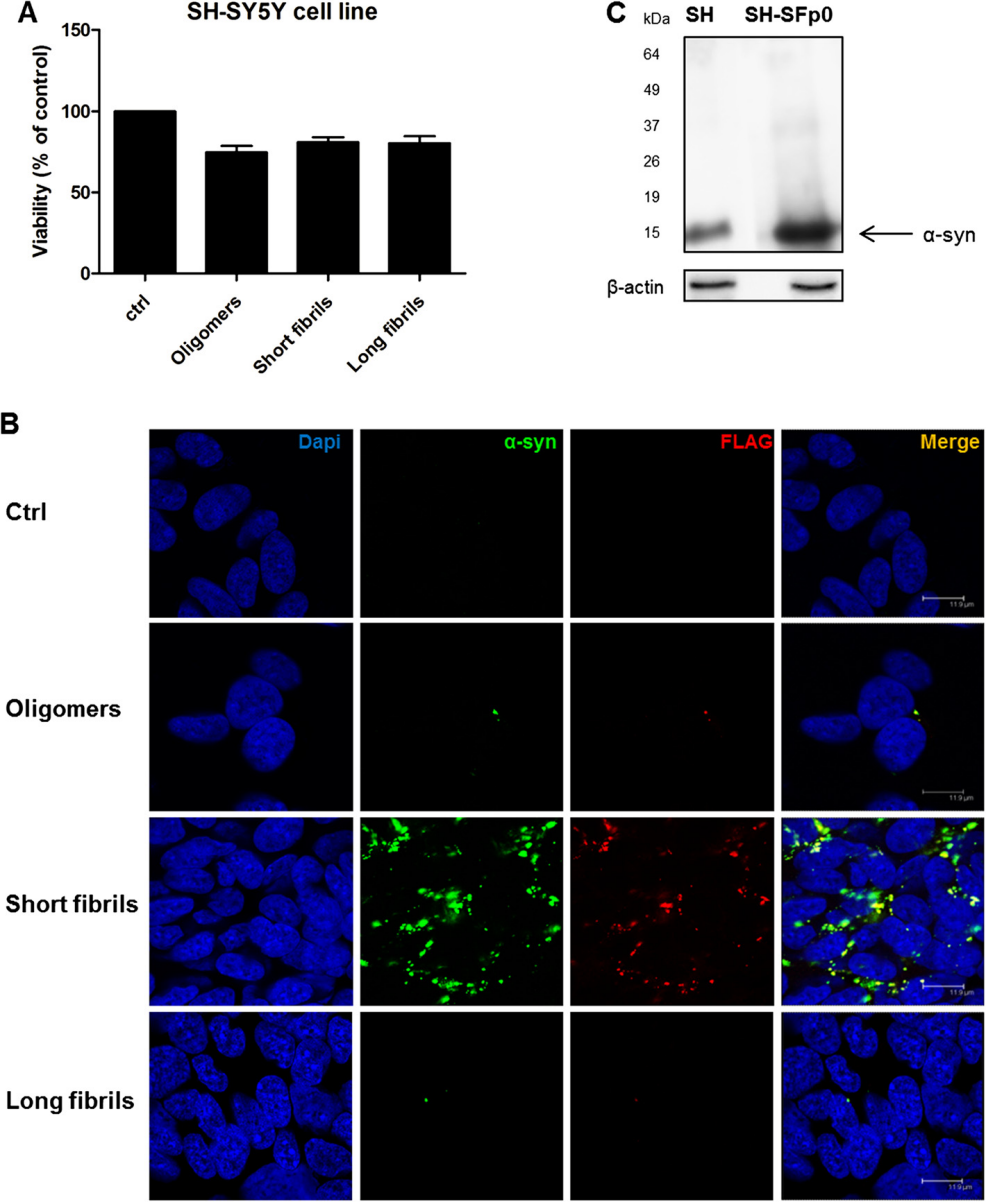
**Cytotoxicity, extracellular α-syn uptake and recruitment of endogenous α-syn in SH-SY5Y cells. (A)** Cells were treated with 5 μg/mL α-syn oligomers, short, and long fibrils for 24 hours. Results are mean ± st. dev. of three independent experiments performed in six replicas. **(B)** Internalization of FLAG-α-syn amyloids into neuroblastoma cells SH-SY5Y. α-Syn deposition (green) was detected by anti-human α-syn antibody. Immunofluorescence was performed on cells exposed to FLAG-α-syn amyloids (oligomers, short amyloid fibrils and long fibrils) for 7 days. Bar, 11.9 μm. **(C)** Formation of endogenous α-syn deposits induced by extracellular α-syn short amyloid fibrils. Cells were treated for 7 days. Western blot analysis was performed using the α-syn (C-20)-R antibody, which recognizes endogenous human α-syn in SH-SY5Y cells. The nuclei (blue) were stained with DAPI. Scale bars, 11.9 μm.

We assessed the cytotoxicity of equal concentrations of three different α-syn preparations on human neuroblastoma SH-SY5Y cells using the 3-(4,5-dimethylthiazol-2-yl)-2,5-diphenyltetrazolium bromide (MTT) assay. This cell survival assay showed that there was no statistically significant difference among the various forms of α-syn preparations in the cell line used (Figure 2A). A comparison of the toxicity of α-syn short and long fibrils does not reflect any specific toxicity, except for the well-known slightly higher toxicity of oligomeric species [22-24] (74.7% viable cells in SH-SY5Y cells).

### Effects of exposure of human neuroblastoma cell lines to different α-syn assemblies

**Figure 3:**
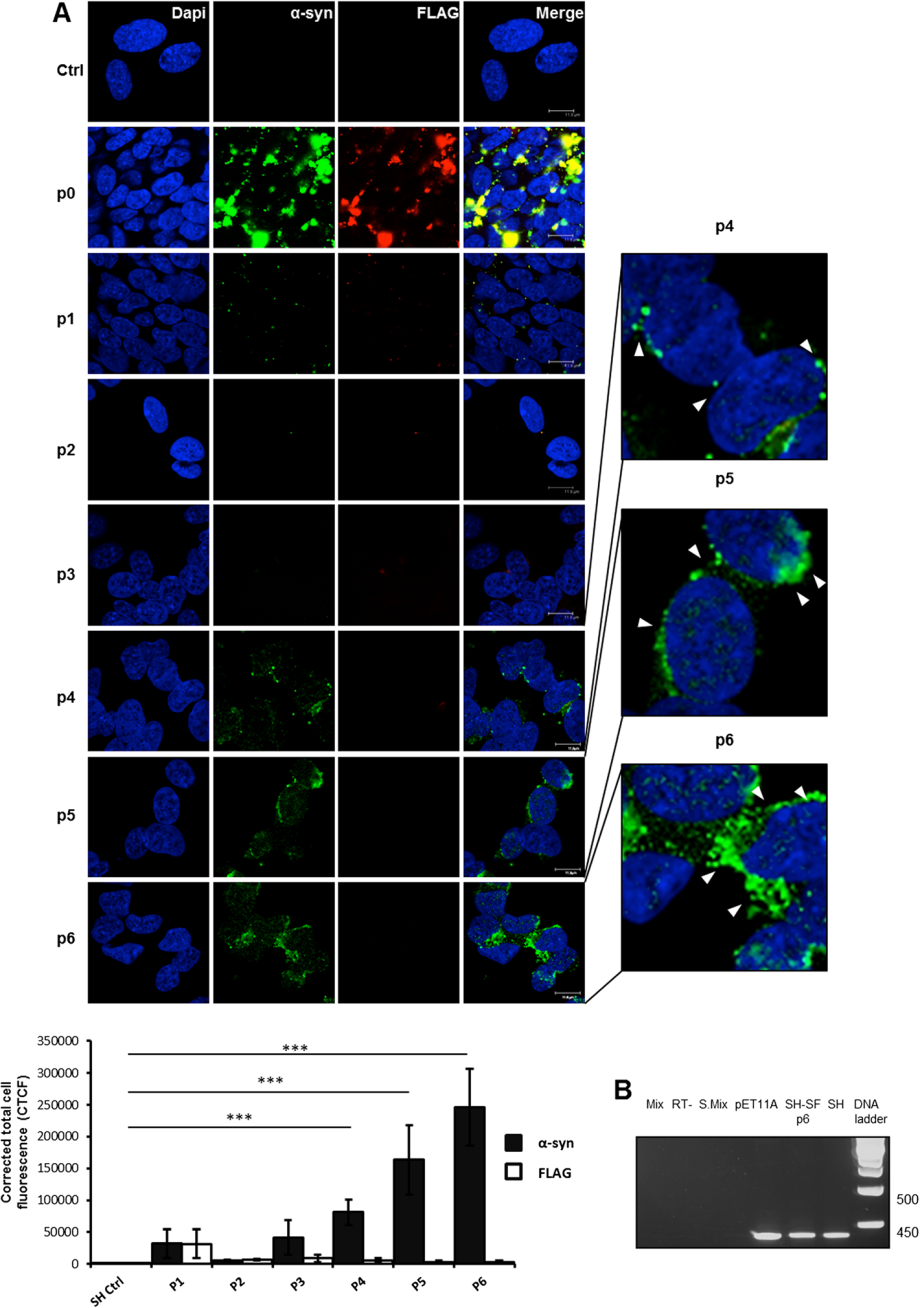
**Infection of the non-transfected SH-SY5Y cells with α-syn short amyloid fibrils and sub-passing over time. (A)** Cells were infected with recombinant human FLAG-α-syn short fibrils. Cells were cultured on coverslips for each passage (p0 to p6). The deposition of exogenous short amyloid fibrils FLAG-α-syn (red) was detected by anti-FLAG antibody. Human endogenous α-syn detected by anti-human α-syn (C-20)-R antibody (green). The nuclei were stained with DAPI (blue). Bar, 12 μm. On the right p4, p5, and p6 zoomed images. Corrected total cell fluorescence (CTCF) from immunofluorescence imaging shows the induction of endogenous α-syn in SH (neuroblastoma SH-SY5Y cell line) infected with human α-syn short amyloid fibrils during the passages (bottom panel). The analysis was performed on at least 150 cells, n = 3, ***p < 0.005. Values are mean ± SD. **(B)** PCR analyses directed to α-syn using the total RNA extracted from non-transfected SH-SY5Y cells. Mix, RT-, S. Mix: negative controls, pET11A: positive control, SH-SF p6: SH-SY5Y cells passaged six times after exposure to short amyloid fibrils, SH: SH-SY5Y cells not treated with short amyloid fibrils. α-Syn was detected after 25 cycles and did not show any significant difference in transcripts before and after infection with short amyloid fibrils of recombinant α-syn.

**Figure 4:**
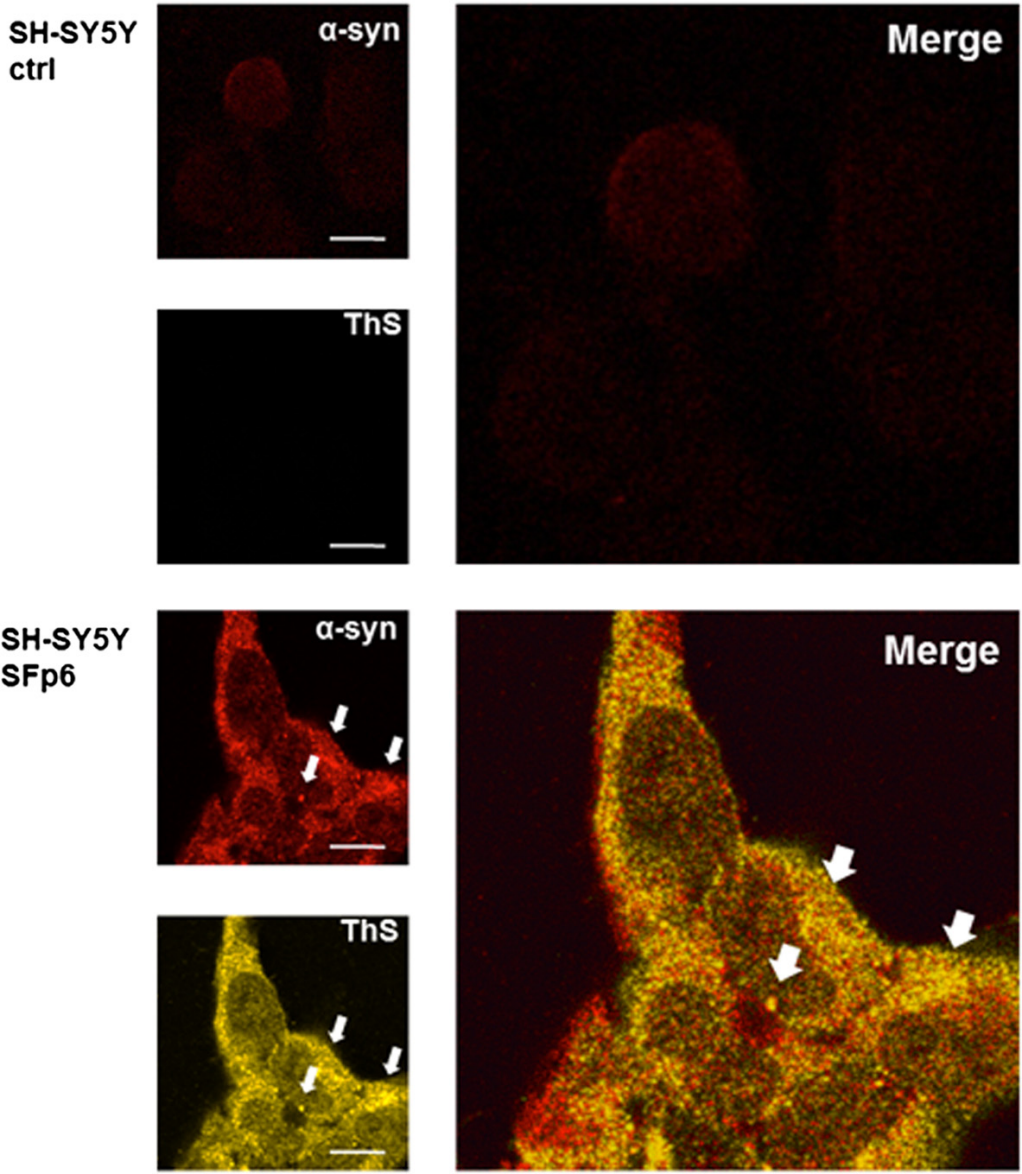
**Thioflavin-S (ThS) positive staining of endogenous α-syn aggregates.** SH-SY5Y cells were infected with recombinant human α-syn short amyloid fibrils (SF: short amyloid fibrils of α-syn) and sub-passed at sixth passage (p6). The deposition and level of α-syn (red) in α-syn-infected cell lines after six passages were detected by anti-α-syn antibody (Additional file 6: Table S2). The colocalization was observed between the ThS signal (yellow) and anti-human α-syn antibody (red). The nuclei (blue) were stained with DAPI. Scale bars, 12 μm.

We adopted different strategies to validate the observation that exogenously added assemblies can enter and be incorporated into cells. Either recombinant human α-syn or FLAG-tagged recombinant human α-syn protein was employed. The use of the FLAG-tagged protein allows the detection of exogenously added protein assemblies. Indeed, after seven days in culture only short amyloid fibrils were able to enter the cells and promote the recruitment of endogenous α-syn into aggregates, while oligomers and long fibrils could not. As a matter of fact, to effectively serve as a seed for aggregation, exogenously added assemblies must first enter the cells and persist for a sufficient period in cellular compartments accessible to the endogenous α-syn protein. A single exposure of β-sheet-rich structures of recombinant human α-syn—which we define as short amyloid fibrils—was sufficient to permit the aggregation of endogenous α-syn in non-transfected human neuroblastoma SH-SY5Y cells. The SH-SY5Y cells exposed to short amyloid fibrils of recombinant human FLAG-tagged α-syn were stained with either an anti-human α-syn antibody or an anti-FLAG immunoglobulin (Figure 2B). The immunoreactivity was mostly cytosolic, with co-localization of anti-FLAG and anti-human α-syn antibody. The result yielded by immunofluorescence was verified by biochemical experiments, which confirmed the increase of endogenous α-syn (Figure 2C).Next, we analyzed the aggregation of the endogenous α-syn in serial cellular passages. For this purpose we incubated the protein preparation with the SH-SY5Y cells only at passage 0 (p0, Figure 3), and at each passage we measured the levels of α-syn using immunofluorescence (Figure 3, graph). Only a residual staining of endogenous α-syn could still be observed in the subsequent passage (p1) and no staining was detectable in the ensuing two passages (p2 and p3). Surprisingly, aggregates of endogenous α-syn appeared at passages four to six (p4-p6). At passage p4 we observed many small, dispersed aggregates, while at passages p5 and p6 we began to observe larger aggregates and perinuclear inclusions in the cytoplasm of SH-SY5Y cells (Figure 3, white arrows). We performed additional passages (up to passage 12) and in each one a sustained aggregation of endogenous α-syn was still present. Therefore, the infected SH-SY5Y cells are able to promote, upon subpassaging, stable aggregation of endogenous α-syn. This accumulation occurs at the protein level since PCR analysis confirmed that the levels of α-syn transcripts were not altered after infection (Figure 3B).Interestingly, the endogenous aggregates of human α-syn in the SH-SY5Y cell line were Thioflavin S (ThS)-positive (Figure 4). This dye binds to β-sheet-rich structures such as amyloids, confirming that the aggregates induced at sixth passage contained significant β-sheets, like LBs do. Thus, the fact that these α-syn aggregates share hallmark features of PD-like LBs led us to conclude that α-syn short amyloid fibrils seed and recruit normal, endogenous α-syn to form pathologic aggregates in the SH-SY5Y cell line.

### Short amyloid fibrils of α-syn induce accumulation earlier in transfected SH-SY5Y cells

**Figure 5:**
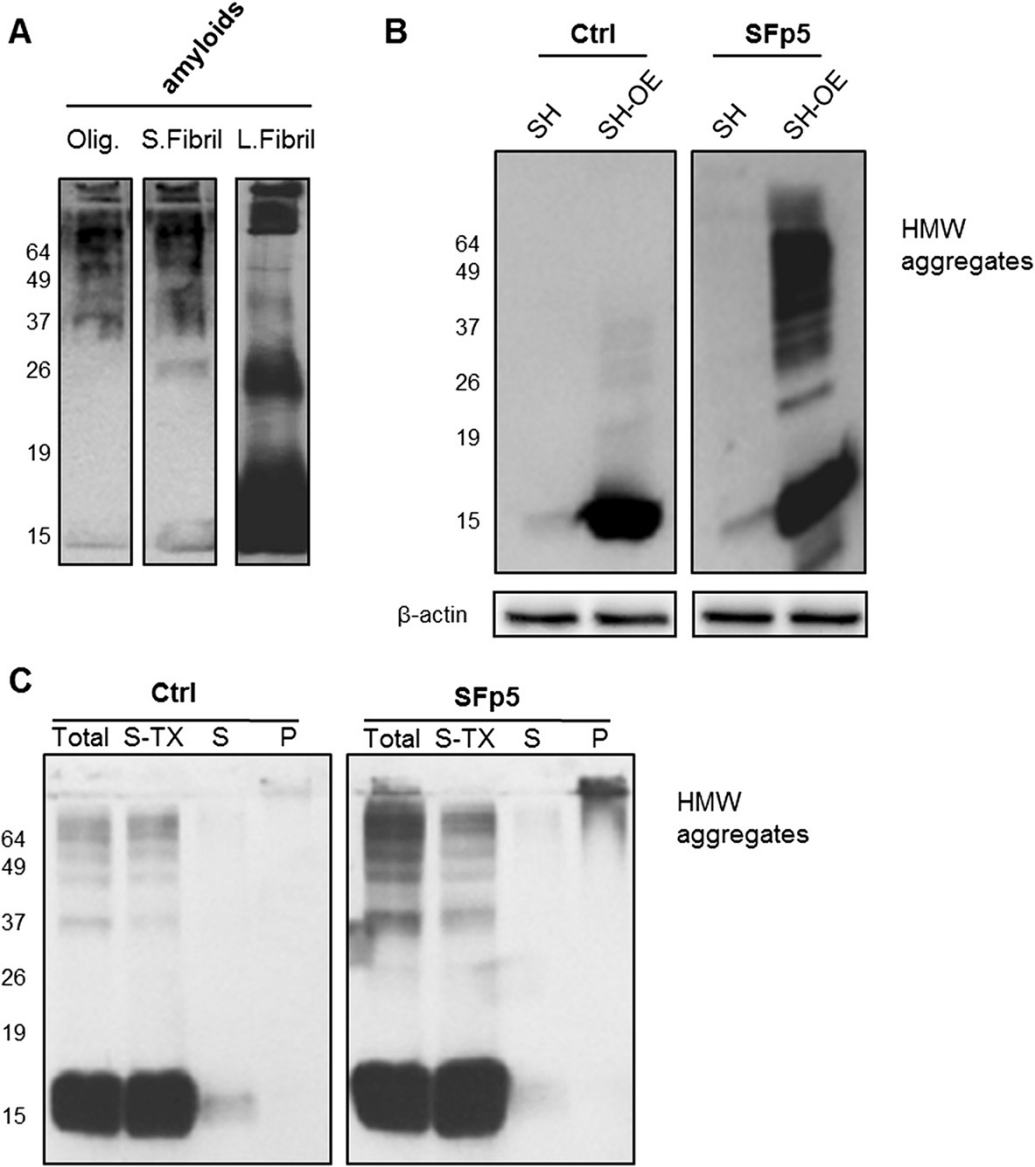
**Aggregation of α-syn in dopaminergic human cell lines infected with recombinant human α-syn short amyloid fibrils and sub-passaged over five passages (p5). (A)** Western blot of different assemblies formed *in vitro*. **(B)** Western blot of cell lysates (SH: SH-SY5Y cells; SH-OE SH-SY5Y overexpressing human α-syn); Ctrl: non-treated cells. SFp5: α-syn short amyloid fibril-infected cells at fifth passage. HMW: High molecular weight aggregates. **(C)** Western blot analysis of aggregated SH-OE cell lysates treated with α-syn short amyloid fibrils, and non-treated (Ctrl); Total: total cell lysates. Sequential extraction of α-syn aggregates in 1% Triton X-100 lysis buffer (S-TX), followed by 2% SDS lysis buffer (S). (P): a pellet fraction after the pellet was treated with 2% SDS. Anti-human α-syn (C-20)-R antibody was used (Additional file 6: Table S2).

We performed similar experiments using a human neuroblastoma SH-SY5Y cell line stably overexpressing human α-syn (Additional file 3: Figure S2). Upon infection with short amyloid fibrils of α-syn and after six passages in cell culture, we observed stronger staining and, most importantly, the presence of several spots of α-syn throughout the cytosol. The accumulation of α-syn was confirmed using the western blot technique at p5 in both SH-SY5Y that overexpress α-syn and the non-transfected cells (Figure 5B). This suggests that α-syn short amyloid fibrils are able to seed aggregation of overexpressed α-syn in this cellular model. Due to the already high levels of α-syn in SH-SY5Y cells that overexpress human wild-type α-syn, the formation of endogenous α-syn aggregates upon infection with recombinant assemblies was evident earlier (p2) compared with non-transfected SH-SY5Y cells (p4) (Additional file 3: Figure S2). Moreover, these aggregates in SH-SY5Y overexpressing α-syn at passage p6 were ThS-positive (Additional file 4: Figure S3). Furthermore, the sequential extraction with 1% Triton X-100 lysis buffer followed by 2% SDS lysis buffer confirmed that the endogenous aggregates of α-syn are insoluble in these buffers. The cells treated with short amyloid fibrils and passaged up to p6 exhibited a significant amount of Triton X-100 insoluble α-syn and high-molecular-weight species (HMW), which required SDS buffer for solubilization (Figure 5C). We observed weak signals in the SDS supernatant fraction. On the contrary, in the pellet fraction we detected bands in the stacking part of the SDS-PAGE gel, which are comparable to those of short amyloid fibrils formed *in vitro* (Figure 5A).

### Endogenous α-syn aggregates display LB-like properties

**Figure 6:**
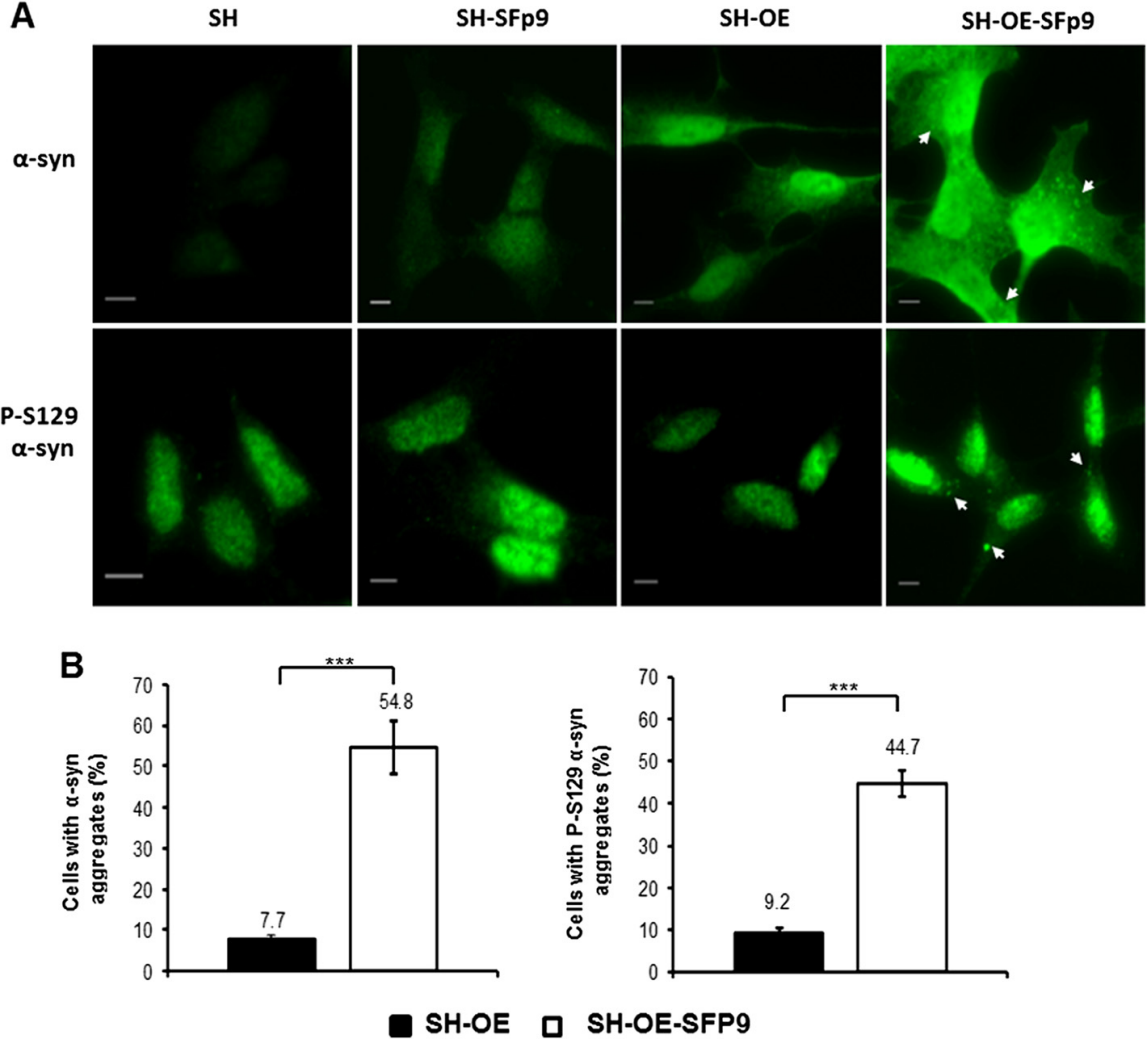
**Short amyloid fibrils induce the formation of phosphorylated α-syn aggregates over passages. (A)** Presence of phosphorylated α-syn aggregates in SH-SY5Y overexpressing α-syn (SH-OE, top left panel). From left to right: neuroblastoma SH-SY5Y (SH), SH-SY5Y infected with human α-syn short amylod fibrils at passage 9 (SH-SFp9), SH-SY5Y overexpressing α-syn (SH-OE) and SH-SY5Y overexpressing α-syn-infected with human α-syn short amylod fibrils at passage 9 (SH-OE-SFp9). Cells were immunolabeled with anti-α-syn antibody (green, upper panel) or anti-phospho S129 α-syn Ab59264 (green, lower panel). Scale bars, 5 μm. Arrows point at α-syn aggregates. Images are representative of at least three independent experiments. **(B)** High percentage of phosphorylated α-syn aggregates in SH-SY5Y α-syn-infected cells. Bar diagram showing quantification of α-syn aggregates (left) and phospho S129 α-syn aggregates (right) in SH-SY5Y α-syn cells and SH-SY5Y α-syn-infected cells. ***p < 0.001 (Student t test, n > 150 cells counted for each condition per experiment). Error bars represent the SEM.

To further characterize endogenous α-syn aggregates, the cell lines were probed with an antibody specific for α-syn phosphorylated at position 129 (P-S129). This post-translational modification of α-syn aggregates has been found in LBs of PD brains. Moreover, P-S129 α-syn has been reported to accumulate in the nuclei of α-syn transfected cells [25], and in Tg mice that express human α-syn [26]. A single-staining immunofluorescence confirmed higher levels of P-S129 α-syn within the nucleus after nine passages upon infection (SH-SFp9 and SH-OE SFp9, Figure 6) in both non-transfected and transfected SH-SY5Y cells, compared to control non-infected cells (Figure 6, lower panel). Since exogenous recombinant short amyloid fibrils are not phosphorylated *in vitro,* these modifications occur *de novo* within the cell after infection and passaging. In addition, the cytoplasm of several α-syn-infected SH-SY5Y cells contained α-syn aggregates (Figure 6, top panel). Quantitative analysis showed that the number of cells with these cytoplasmic inclusions was remarkably higher in amyloid-infected cells compared to non-infected cells (54.8% vs. 7.7%, p < 0.001) (Figure 6B, bottom left). Thus, we concluded that although overexpression of α-syn induces aggregation, this occurs to a much greater extent when combined with infection. Interestingly, in amyloid-infected cells we found the same increase in phosphorylated aggregates compared to transfected cells (44.7% vs. 9.2%, p < 0.001) (Figure 6B, bottom right) indicating that most α-syn aggregates induced by short amyloid fibrils are phosphorylated.

Together, these observations indicate that the endogenous α-syn aggregates in this cell culture model exhibit LB-like defining features.

## Discussion

Immortalized cell lines and primary-cultured cells are commonly used in the research of synucleinopathies. Volpicelli-Daley *et al.*[16] have reported a seeded inclusion formation in primary neuronal cultures that did not overexpress α-syn, and Sacino *et al.*[27] have investigated the aggregation process in primary mixed neuronal-glial cultures using recombinant wild-type α-syn and PD-linked mutations (A53T and E46K). Nevertheless, the drawback of employing primary cell models is that they have a limited use for transmission experiments. Moreover, the cellular populations might be altered during repeated sub-passaging. This is one reason why we focused on immortalized cell lines, as they yield more homogenous cell preparations, cells can be passaged for long periods, and they harbor a sufficient quantity of samples for biochemical analyses. Indeed, here we show that one defined molecular assembly of extracellular α-syn is able to induce endogenous α-syn to aggregate, thus supporting the hypothesis that α-syn pathology can spread *via* a prion-like self-templating mechanism. This finding is a shared feature with other cell culture models that are permissive to prion replication, such as neuroblastoma N2a cells [28]. This infected cell culture model has provided valuable insights into the biogenesis of the scrapie prion protein (PrP^Sc^) in terms of subcellular localization, conversion, and physiopathological consequences. Therefore, using the immortalized SH-SY5Y cell line, which replicates α-syn aggregates, is important to better understand the mechanism involved in α-syn aggregation. We focused our attention on non-transfected SH-SY5Y cells, and these findings were also confirmed in another cell line that does not overexpress α-syn protein. The GT1 cell line [29] was treated with short amyloid fibrils of recombinant human α-syn, and we observed the same results, indicating that this aggregation phenomenon extends to multiple cell types, even to those that do not overexpress α-syn (Additional file 5: Figure S4). These cell cultures will allow us to study the mechanism of cellular uptake of fibrils (p0), the conversion mechanism of endogenous α-syn protein into aggregates (from p4), and their sub-cellular localization.

## Conclusions

In this study we characterized three different fibril assemblies of recombinant α-syn protein (oligomers, short fibrils, and long fibrils) and evaluated their effect on immortalized cell lines. Our findings show that recombinant human α-syn can adopt a conformation (i.e., short amyloid fibrils) able to recruit cellular α-syn into aggregates that can replicate and accumulate *in vitro* in non-transfected cells. Although we used synthetic recombinant assemblies, and not the aggregates from brain homogenates either from PD patients or transgenic mice, we observed prolonged and sustained aggregation and accumulation of endogenous α-syn in non-transfected and transfected SH-SY5Y cell lines. Our findings point to α-syn short amyloid fibrils as the pathological species of α-syn aggregates primarily involved in the transmission of α-syn pathology. Our data support the hypothesis that the sequence of events leading to LB formation can be recapitulated in cultured cells. In summary, we developed a new approach for the study of α-syn aggregates in cell culture. The advantage of our system is that it can be employed for prolonged experimental periods since the accumulation of α-syn is sustained over time. This approach systematically minimizes the possibility of clonal artifacts and represents a valid tool for potential therapeutic screening that targets pathologic α-syn aggregates.

## Methods

### Cell lines

The GT1-1 cells were obtained from the laboratory of Professor Krister Kristensson, Department of Neuroscience, Karolinska Institutet Stockholm, Sweden, following an MTA agreement with Dr. Pamela Mellon of the University of California, San Diego, USA. This line is often used in prion biology and it is established from gonadotropin hormone-releasing neurons immortalized by genetically targeted tumorigenesis in transgenic mice [29]. The SH-SY5Y cell line is a thrice-cloned sub-line of SK-N-SH cells, which were originally established from a bone marrow biopsy of a neuroblastoma patient with sympathetic adrenergic ganglial origin [30]. GT1 cells were seeded in 10-cm plates containing 10 mL of Dulbecco’s modified Eagle’s medium (DMEM) culture media, supplemented with 10% fetal bovine serum (FBS) and 1% penicillin-streptomycin. SH-SY5Y normal cells were cultivated in 10-cm plates, containing 10 mL of minimal EMEM: Ham F12 (1:1) culture media, supplemented with 15% FBS, 1% non-essential amino acids, 0.5% L-glutamax, 1% penicillin-streptomycin and 1% G-418 (only for SH-SY5Y wild-type cells transfected for overexpressing α-syn). The cells were grown at 37°C in 5% CO_2_ to 95% confluence for 1 week before splitting at 1:10 for further cultivation.

### Cytotoxicity assay

The cytotoxic effect of α-syn fibrils was assessed by measuring cellular redox activity with 3-(4,5-dimethylthiazol-2-yl)-2,5-diphenyltetrazolium bromide (MTT). Briefly, 100,000 SH-SY5Y and GT1 cells/well were cultured in a 96-well microtiter plate and treated with α-syn oligomers, short fibrils and long fibrils (suspended by vortexing). Following a 24-h incubation, the cytotoxic effect was assessed by measuring cellular redox activity, following the manufacturer’s (Sigma) instructions.

### Expression and purification of recombinant human α-syn

Expression and purification of recombinant human α-syn were performed in accordance with the method previously described [30]. Briefly, the human α-syn gene was cloned and expressed in pET11a vector using BL21 (DE3) *E. coli* strain. Expression of α-syn was obtained by growing cells in Luria-Bertani broth medium with 100 mg/mL ampicillin at 37°C until an O.D._600_ of about 0.6, followed by induction with 0.6 mM isopropyl β-D-thiogalactoside (IPTG) for 5 hours. The protein was purified according to the method of Huang *et al.*[31].

### α-syn amyloid preparations

All solutions were sterilized by filtration through a 0.22-μm filter prior to each assay run. Reactions were prepared in a 96-well black plate (BD Falcon), and each well contained 200 μL of reaction solution [1.5 mg/mL recombinant human α-syn, 100 mM NaCl, 10 μM Thioflavin T (ThT) in 20 mM Tris–HCl pH 7.4]. Each sample analysis was performed in fifteen replicates. Each well contained one 3-mm glass bead (Sigma). The plate was covered with sealing tape (Fisher Scientific), incubated at 37°C under continuous shaking, and read on SpectraMax M5 fluorescence plate reader (Molecular Devices) by top fluorescence reading every 5 min at excitation of 444 nm and emission of 485 nm.

### AFM analysis

AFM analysis was performed in accordance with the method previously described [32]. Three to five μL of fibril solution was deposited onto a freshly cleaved piece of mica and left to adhere for 30 min. Samples were then washed with distilled water and blow-dried under a flow of nitrogen. Images were collected at a line scan rate of 0.5-2 Hz in ambient conditions. The AFM free oscillation amplitudes ranged from 25 nm to 40 nm, with characteristic set points ranging from 75% to 90% of these free oscillation amplitudes. AFM data were analyzed with Gwyddion (gwyddion.net) and SPIPTM (http://www.imagemet.com) software.

### α-syn amyloid solution for cell infection

The α-syn amyloid solution was transferred from the 96-well plate into a 1.5 mL Eppendorf tube and collected under sterile conditions. The solution was ultracentrifuged at 100,000 g for 30 min at 4°C (Beckman Coulter). Pellets were resuspended in 1X PBS and then sonicated (Branson 2510) for 5 min prior to adding to the cultured cell plate.

### α-syn fibril infection in cell lines

Three hundred μg of α-syn amyloids was added to GT1 and SH-SY5Y cell plates (10 cm-plate) and exposed in the cell culture media for 7 days before the next splitting and media change. Cells were split and maintained for six additional passages. Cell lysates were collected at each passage for Western blotting and immunofluorescence studies. For immunofluorescence of amyloids internalization, cells were cultured in 12-well plates with coverslips; 30 μg of α-syn amyloids was added to the cell culture media and incubated for 7 days.

### Detection of α-syn aggregates in infected cells and analysis by Western blotting

The total protein content of samples was measured by bicinchoninic acid assay (BCA) (Pierce). Fifty μg of cell lysates protein was used and 5X loading buffer was added in a 1:5 ratio. The samples were boiled for 5 min at 100°C, loaded onto a 10% Tris-Glycine SDS-PAGE gel, and transferred overnight onto Immobilon P PVDF membranes (Millipore). Membranes were blocked by 5% nonfat milk, incubated with 0.4 μg/mL rabbit polyclonal anti-α-syn antibody (Santa Cruz) followed by incubation with goat anti-rabbit IgG F(ab)2 fragment conjugated with horseradish peroxidase. Blots were developed with the enhanced chemiluminescent system (ECL, Amersham Biosciences) and visualized on Hyperfilm (Amersham Biosciences). To analyze α-syn aggregation in cell samples, cells were scraped in TBS buffer containing 1% Triton X100, protease cocktail inhibitors and phosphatase inhibitor. After sonication (Sonicrep 150) with 10 amplitude microns for 3 times (30 seconds of sonication and 30 seconds intermediate stop) cells were centrifuged at 2000 RPM for 5 min before the protein was quantified. Fifty μg of sample was centrifuged at 100,000 g for 30 min. Supernatant (in term S-TX) was collected and the pellets were resuspended in 2% SDS. Samples in 2% SDS were centrifuged, supernatant (S) and pellet (P) were collected. All the fractions were added to loading buffer 5X in ratio 1:5 and prepared similarly for Western blotting.

### Reverse transcribed–polymerase chain reaction

Total RNA was extracted from cultured cells using the Trizol® Reagent (Life Technologies) extraction method. From 1 μg of total RNA, cDNA was synthesized using 200 units MoMuLV-reverse transcriptase (SuperScrip™ III RT; Life Technologies), oligo (dT) primers and 1 μl 10 mM dNTP Mix in a final volume of 13 μl. Ten microliters of the 25-fold diluted solution was subjected to quantitative PCR analysis. The following primer pairs were used for α-syn: Hu_Syn_FW 5′-ATGGATGTATTCATGAAA-3′, Hu_Syn REV 5′-TTAGGCTTCAGGTTCGTA-3′. PCR amplification was conducted using the Phusion® High-Fidelity DNA Polymerase protocol, under the following conditions: initial denaturation at 98°C for 30s, 10s at 98°C, 30s at 45°C and 30s at 72°C for 25 cycles, followed by 10 min at 72°C. After amplification, 10 μl aliquots were electrophoresed in 1.5% agarose gel, followed by photographic recording of the gel stained with ethidium bromide.

### Immunocytochemistry and ThS staining of α-syn fibril-infected cells

Cells on coverslips were washed with PBS and fixed with 4% paraformaldehyde, then washed twice, 15 min/time with PBS and blocked in blocking buffer [5% normal goat serum (NGS) in PBS + 0.3% Triton] for 1 hour. For Thioflavin S (ThS) staining, fixed cells were incubated with 0.025% of the fluorophore (Sigma) for 8 min and washed three times with 80% ethanol for 5 min each time, prior to the antibody incubations. Fluorescence immunocytochemistry was performed using the primary and secondary antibodies listed in Additional file 6: Table S2. Primary antibodies were made up in 1% blocking buffer and PBS. After incubation, the cells were washed 5 times for 5 min/time with PBS; secondary antibodies were incubated in 1% blocking buffer and PBS for 45 min. Finally, cells were washed 5 times, 5 min/time with PBS, and counterstained with DAPI to reveal nuclei, then mounted in Vectashield Mounting Medium. Cell coverslips were stored at 4°C for confocal fluorescence microscopy. The fluorescence was measured in at least 10 randomly chosen observation fields for each experimental condition using Leica SP5 confocal laser-scanning microscope. The increase in fluorescence was measured calculating CORRECTED TOTAL CELL FLUORESCENCE (CTCF) using the formula: CTCF = Integrated density – (Area of selected cell X Mean fluorescence of Background readings) using the ImageJ 1.47 Software (NIH). Quantification experiments were carried out independently at least three times; more than 150 cells were counted for each condition. Individual differences were assessed using individual student’s t-tests in GraphPad Prism software (San Diego, CA). Data are shown as mean ± standard deviation (SD).

### Immunofluorescence microscopy analysis of phosphorylated aggregates

All dopaminergic neuroblastoma SH-SY5Y cells were grown on ibidi dishes (Biovalley) for microscopy. After washing in PBS, cells were fixed using 4% (w/v) paraformaldehyde (Sigma) in PBS for 15 min and permeabilized with 0.01% Triton X-100 (Sigma) in PBS for 3 min, washed and blocked in 2% Bovine serum albumin (Sigma) in PBS for 20 minutes. Cells were immunostained using anti-α-syn (Santa Cruz Biotechnology, INC) and anti-phospho S129 α-syn (Abcam) followed by secondary antibody coupled to Alexa 488 (Invitrogen). Cells were mounted in Aqua Poly/Mount (Polysciences) and pictures were acquired using white field Axiovision microscope (Zeiss) with a 63x objective. Quantification experiments were carried out independently at least three times; more than 150 cells were counted for each condition. Individual differences were assessed using individual student’s t-tests in GraphPad Prism software (San Diego, CA). Data are shown as mean ± standard error of the mean (SEM).

## Abbreviations

α-syn: α-synuclein; LBs: Lewy bodies; PD: Parkinson disease; AD: Alzheimer disease; DLB: Dementia with lewy bodies; MSA: Multiple system atrophy.

## Competing interests

The authors declare that they have ho competing interests.

## Authors’ contributions

SA and TTNL contributed to design the study, performed most experiments, analyzed the data, performed the statistical analysis, and helped draft the manuscript. SC performed the AFM experiments and analyzed the data. SAb performed the P-S129 immunofluorescence experiment. LC and CZ contributed to the design and coordination of the study. FM and FT helped conceive the study, participated in its design and coordination and helped draft the manuscript. SG provided the human cell lines used in this work. GL conceived, designed and coordinated the study, analyzed the data, and drafted the manuscript. All authors read and approved the final manuscript.

## Supplementary Material

Additional file 1: Figure S1 Plasmids used for expressing human α-syn and FLAG-human α-syn in E. coli BL21(DE)3. (A) Homology comparison between mouse α-syn and human α-syn, (B) highlighting amino acid substitutions. (C) Typical chromatogram obtained from human α-syn protein purification by anion-exchange chromatography with HiTrap Q Sepharose Fast Flow column. The protein was eluted with a 0–0.5 M NaCl gradient in 20 mM Tris pH8.0. Fractions B7-B2 correspond to purified protein. (D) Expression of recombinant human α-syn protein, 15% SDS-PAGE. Lane 1 and 5, molecular mass marker; lane 2, whole cell extract before IPTG induction; lanes 3 and 4, cell extract after IPTG induction: 5 hours (lane 3), overnight induction (lane 4); lane 6, purified α-syn protein. Click here for file

Additional file 2: Table S1 Summary of dimensions of the three α-syn amyloid preparations. Click here for file

Additional file 3: Figure S2 Infection of the transfected SH-SY5Y cells with α-syn short amyloid fibrils and sub-passing over time. The SH-SY5Y cells that stably overexpress human wild-type α-syn (SH-OE) were infected with recombinant human FLAG-α-syn short fibrils. Cells were cultured on coverslips for each passage (P0 to P6). The deposition of exogenous short amyloid fibrils FLAG-α-syn (red) was detected by anti-FLAG antibody. Human endogenous α-syn detected by anti-human α-syn (C-20)-R antibody (green). The nuclei were stained with DAPI (blue). Bar, 12 μm. Corrected total cell fluorescence (CTCF) from immunofluorescence imaging shows the induction of endogenous α-syn in SH (neuroblastoma SH-SY5Y cell line) infected with human α-syn short amyloid fibrils during the passages (bottom panel). The analysis was performed on at least 150 cells, n = 3, ***p < 0.005. Values are mean ± SD. Click here for file

Additional file 4: Figure S3 Thioflavin-S (ThS) positive staining of endogenous α-syn aggregates in stably transfected SH-SY5Y cell line. The SH-SY5Y cells that overexpress wild-type human α-syn (SH-OE) were infected with recombinant human α-syn short amyloid fibrils (SF: short amyloid fibrils of α-syn) and sub-passaged at sixth passage (p6). The deposition and level of α-syn (red) in α-syn-infected cell lines after six passages were detected by anti-α-syn antibody (Additional file 6: Table S2). The colocalization was observed between the ThS signal (yellow) and anti-α-syn antibody (red). The nuclei (blue) were stained with DAPI. Scale bars, 12 μm. Click here for file

Additional file 5: Figure S4 The treatment with short amyloid fibrils in another cell line (GT1 cells) induces the same behavior. (A) Immunofluorescence images show the induction of endogenous mouse α-syn in GT1 cells after infection with human α-syn short amyloid fibrils over four passages.The detection of exogenously added short amyloid fibrils was performed using an anti human α-syn antibody [LB 509] (red). Mouse endogenous α-syn was detected by anti mouse α-syn antibody D37A6 (green). The nuclei were stained with DAPI (blue). Bars of Ctrl, p0 and p1 are 12 μm; bars of p2, p3, p4 are 24 μm. The graph shows the analysis of the relative corrected total cell fluorescence (CTCF) of endogenous α-syn in GT1 cells infected with human α-syn short amyloid fibrils during the passages. The analysis was performed on at least 150 cells, n = 3, ***p < 0.005. Values are mean ± SD. (B) ThS staining of endogenous aggregated in GT1 cells. (C) Western blotting of cell lysates exposed to anti-α-syn antibody; SFp5: human α-syn short amyloid infected cells at fifth passage. Click here for file

Additional file 6: Table S2 Antibodies used in this study. Click here for file
