# Supplementary figures and images for: Defined α-synuclein prion-like molecular assemblies spreading in cell culture
Source: BMC Neurosci. 2014 Jun 4;15:69. doi: 10.1186/1471-2202-15-69 (PMC4064824; doi:10.1186/1471-2202-15-69)

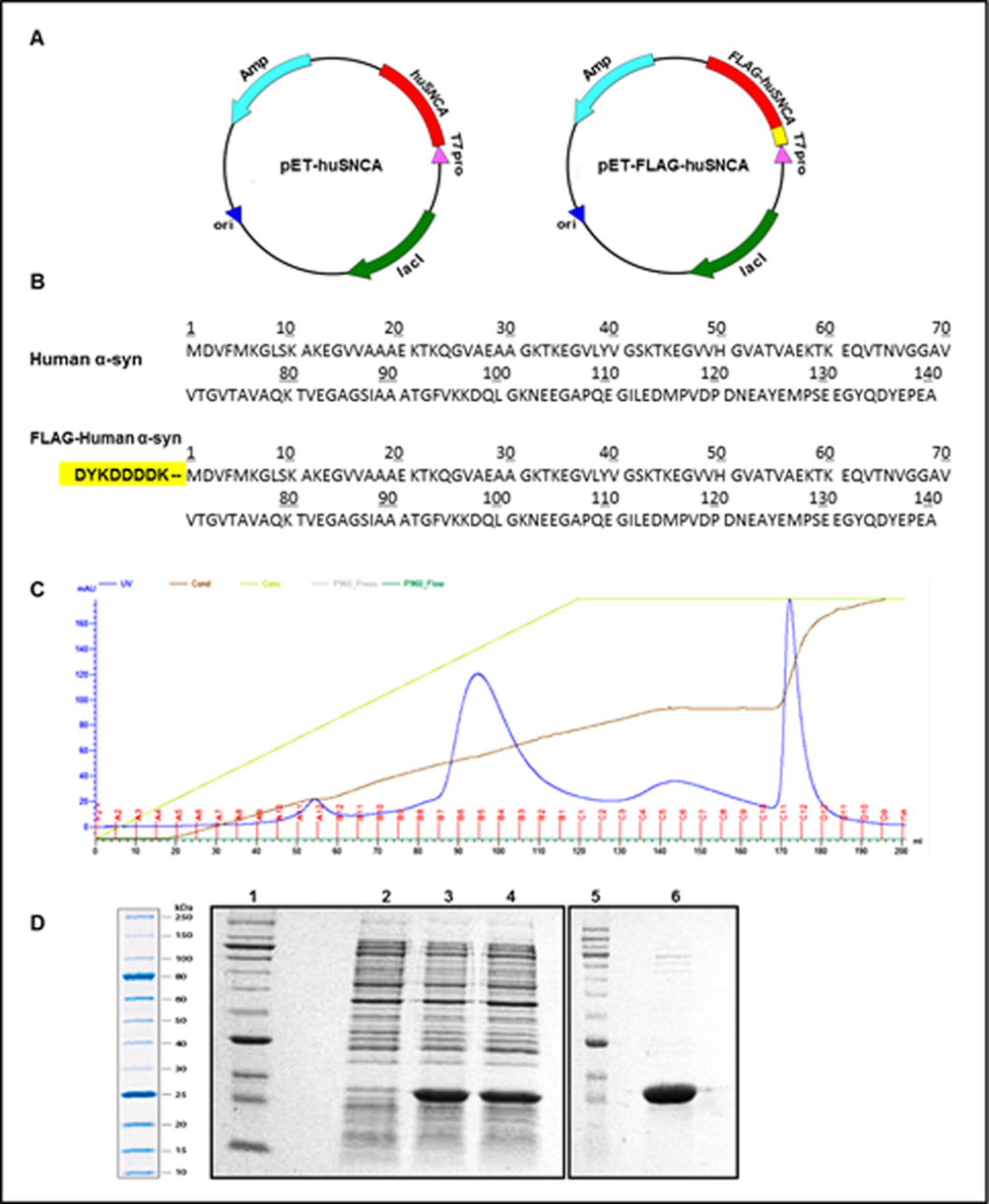

Supplement: Additional file 1: Figure S1 — Plasmids used for expressing human α-syn and FLAG-human α-syn in E. coli BL21(DE)3. (A) Homology comparison between mouse α-syn and human α-syn, (B) highlighting amino acid substitutions. (C) Typical chromatogram obtained from human α-syn protein purification by anion-exchange chromatography with HiTrap Q Sepharose Fast Flow column. The protein was eluted with a 0–0.5 M NaCl gradient in 20 mM Tris pH8.0. Fractions B7-B2 correspond to purified protein. (D) Expression of recombinant human α-syn protein, 15% SDS-PAGE. Lane 1 and 5, molecular mass marker; lane 2, whole cell extract before IPTG induction; lanes 3 and 4, cell extract after IPTG induction: 5 hours (lane 3), overnight induction (lane 4); lane 6, purified α-syn protein. [file 1471-2202-15-69-S1.tiff]

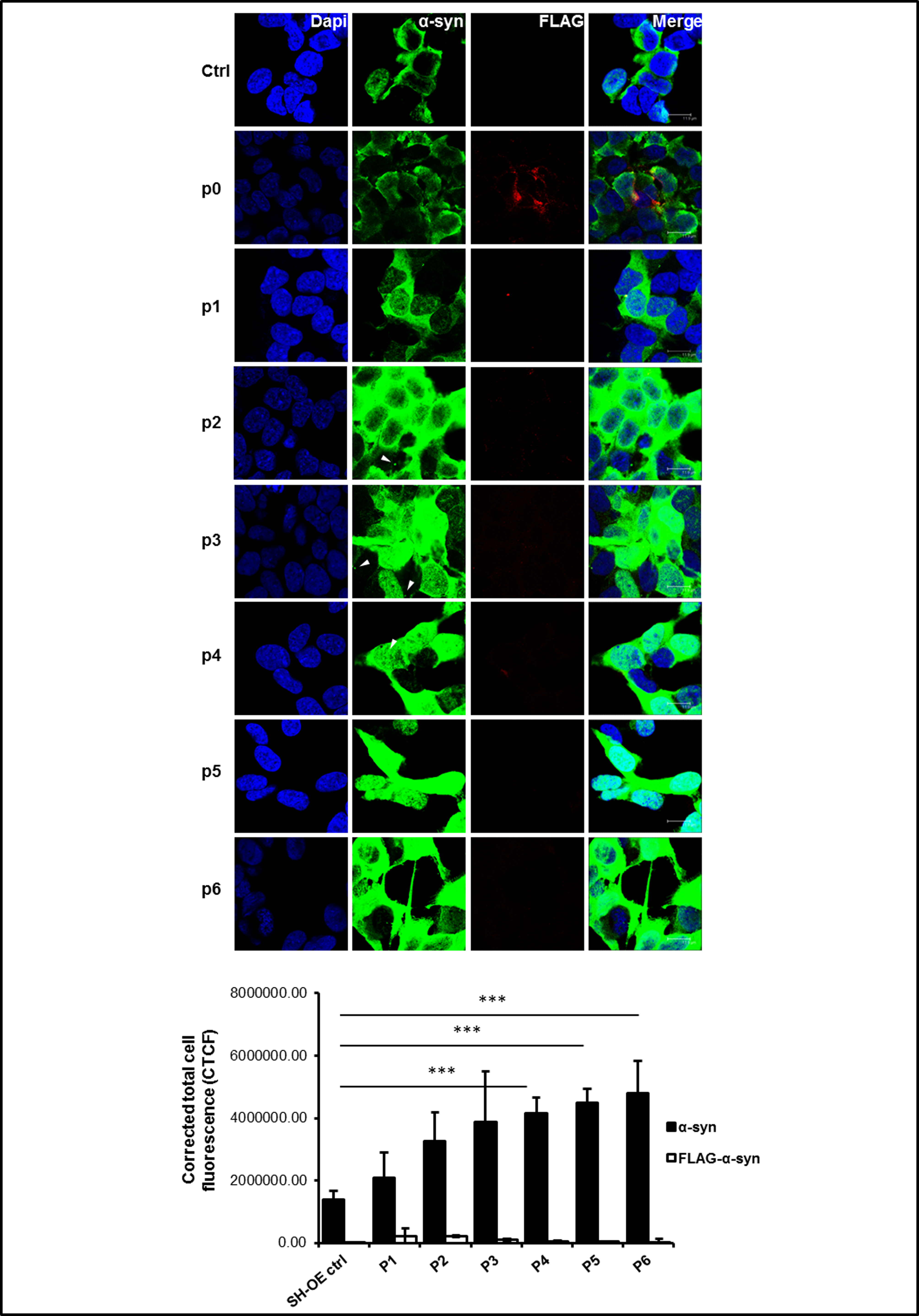

Supplement: Additional file 3: Figure S2 — Infection of the transfected SH-SY5Y cells with α-syn short amyloid fibrils and sub-passing over time. The SH-SY5Y cells that stably overexpress human wild-type α-syn (SH-OE) were infected with recombinant human FLAG-α-syn short fibrils. Cells were cultured on coverslips for each passage (P0 to P6). The deposition of exogenous short amyloid fibrils FLAG-α-syn (red) was detected by anti-FLAG antibody. Human endogenous α-syn detected by anti-human α-syn (C-20)-R antibody (green). The nuclei were stained with DAPI (blue). Bar, 12 μm. Corrected total cell fluorescence (CTCF) from immunofluorescence imaging shows the induction of endogenous α-syn in SH (neuroblastoma SH-SY5Y cell line) infected with human α-syn short amyloid fibrils during the passages (bottom panel). The analysis was performed on at least 150 cells, n = 3, ***p < 0.005. Values are mean ± SD. [file 1471-2202-15-69-S3.tiff]

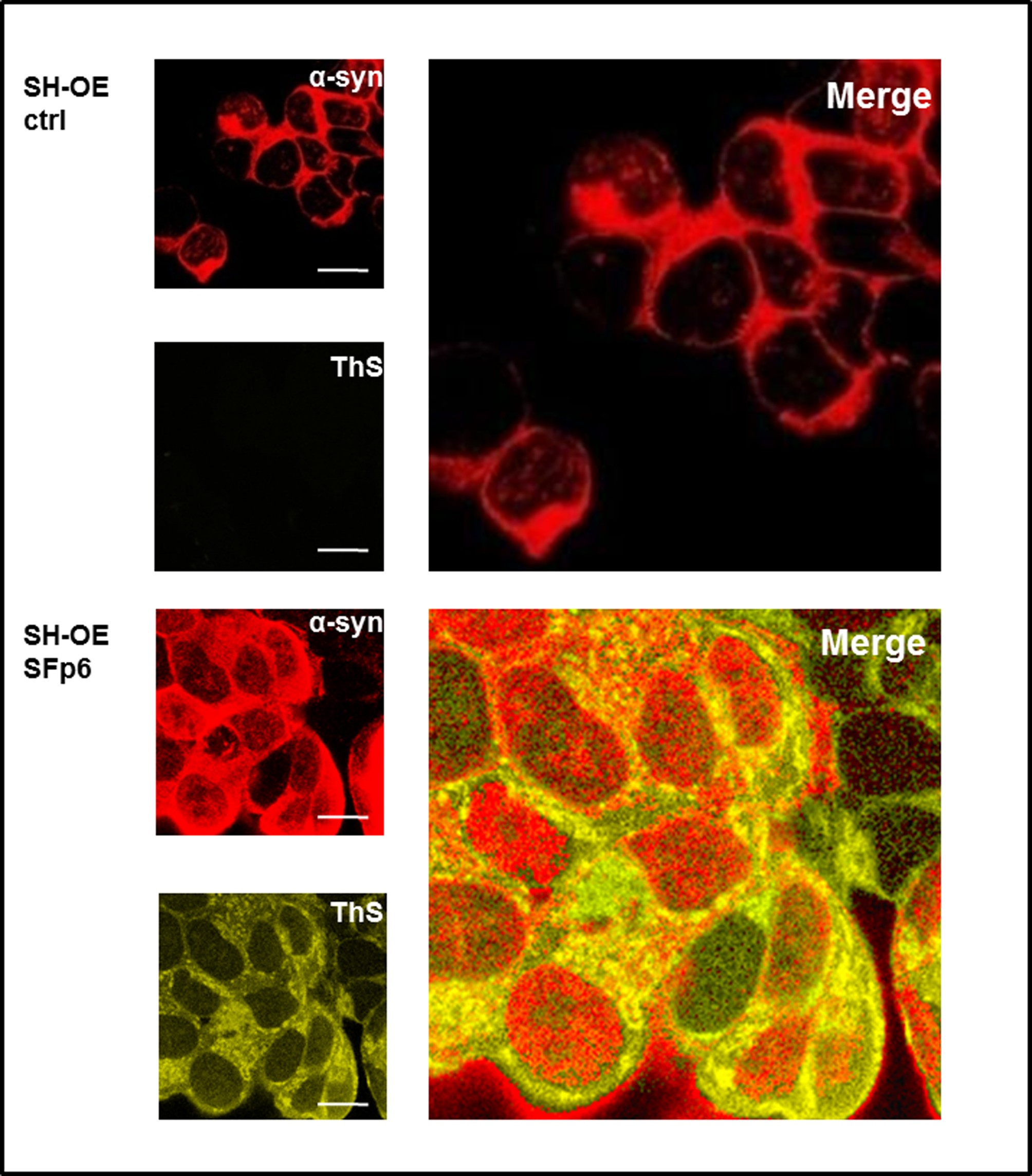

Supplement: Additional file 4: Figure S3 — Thioflavin-S (ThS) positive staining of endogenous α-syn aggregates in stably transfected SH-SY5Y cell line. The SH-SY5Y cells that overexpress wild-type human α-syn (SH-OE) were infected with recombinant human α-syn short amyloid fibrils (SF: short amyloid fibrils of α-syn) and sub-passaged at sixth passage (p6). The deposition and level of α-syn (red) in α-syn-infected cell lines after six passages were detected by anti-α-syn antibody (Additional file 6: Table S2). The colocalization was observed between the ThS signal (yellow) and anti-α-syn antibody (red). The nuclei (blue) were stained with DAPI. Scale bars, 12 μm. [file 1471-2202-15-69-S4.tiff]

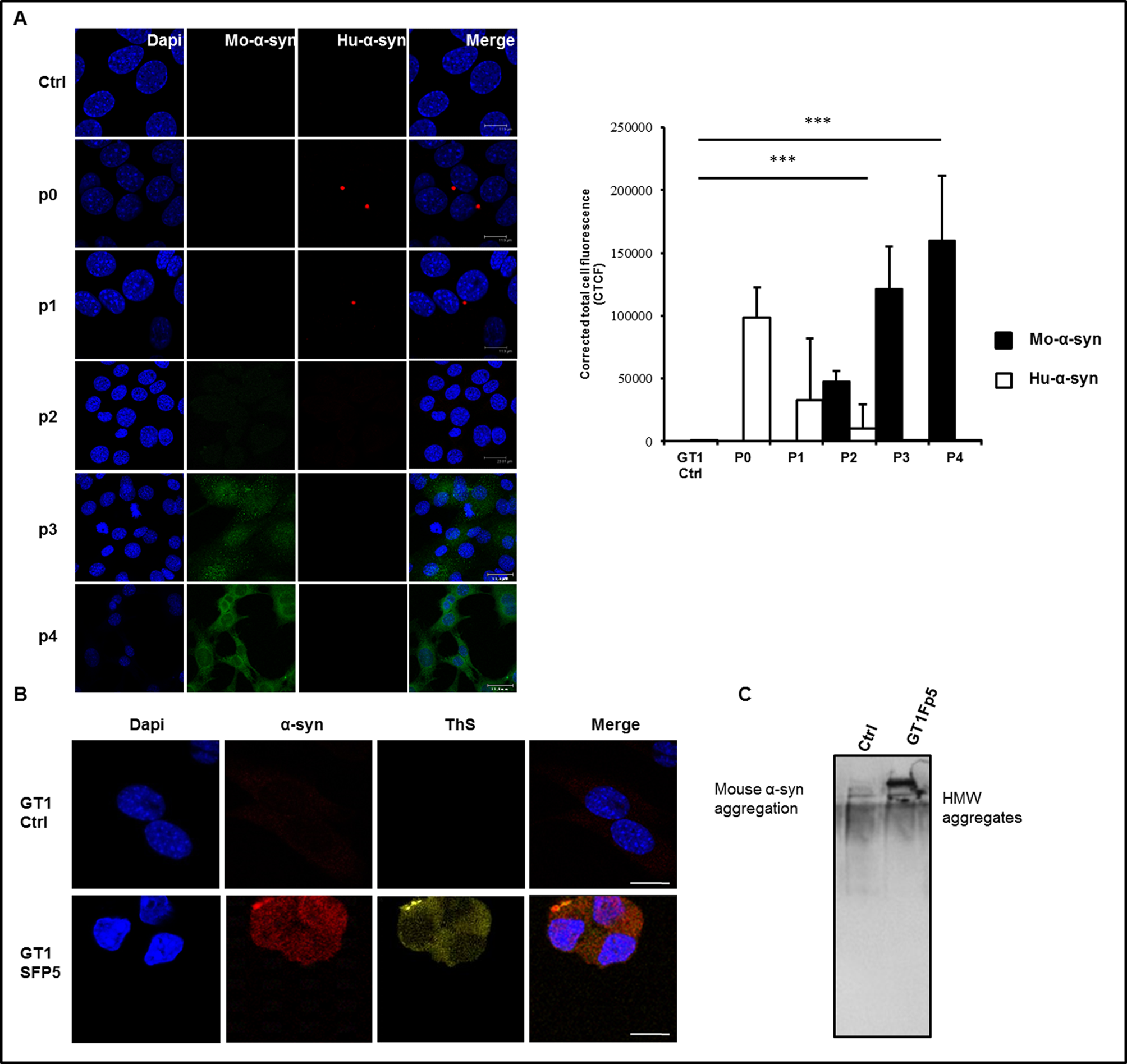

Supplement: Additional file 5: Figure S4 — The treatment with short amyloid fibrils in another cell line (GT1 cells) induces the same behavior. (A) Immunofluorescence images show the induction of endogenous mouse α-syn in GT1 cells after infection with human α-syn short amyloid fibrils over four passages.The detection of exogenously added short amyloid fibrils was performed using an anti human α-syn antibody [LB 509] (red). Mouse endogenous α-syn was detected by anti mouse α-syn antibody D37A6 (green). The nuclei were stained with DAPI (blue). Bars of Ctrl, p0 and p1 are 12 μm; bars of p2, p3, p4 are 24 μm. The graph shows the analysis of the relative corrected total cell fluorescence (CTCF) of endogenous α-syn in GT1 cells infected with human α-syn short amyloid fibrils during the passages. The analysis was performed on at least 150 cells, n = 3, ***p < 0.005. Values are mean ± SD. (B) ThS staining of endogenous aggregated in GT1 cells. (C) Western blotting of cell lysates exposed to anti-α-syn antibody; SFp5: human α-syn short amyloid infected cells at fifth passage. [file 1471-2202-15-69-S5.tiff]
