# Supplementary material for: Defined α-synuclein prion-like molecular assemblies spreading in cell culture
Source: BMC Neurosci. 2014 Jun 4;15:69. doi: 10.1186/1471-2202-15-69 (PMC4064824; doi:10.1186/1471-2202-15-69)
Supplement: Additional file 2: Table S1 — Summary of dimensions of the three α-syn amyloid preparations. [file 1471-2202-15-69-S2.docx]

### Table S1. Summary of dimensions of the three α-syn amyloid preparations.

| α-Syn  preparations | Height (nm) | St. Dev (h) | Length (μm) | St. Dev (L) |
| --- | --- | --- | --- | --- |
| Oligomers | 0.654 | 0.113 | 0.006 | 0.00172 |
| Short fibrils | 2.794 | 1.204 | 1.283 | 0.95824 |
| Long fibrils | 6.080 | 1.376 | 5.053 | 1.99995 |
