# Supplementary material for: Defined α-synuclein prion-like molecular assemblies spreading in cell culture
Source: BMC Neurosci. 2014 Jun 4;15:69. doi: 10.1186/1471-2202-15-69 (PMC4064824; doi:10.1186/1471-2202-15-69)
Supplement: Additional file 6: Table S2 — Antibodies used in this study. [file 1471-2202-15-69-S6.docx]

**Table S2.** Antibodies used in this study

| Antibody | Source | Host | Dilution (IF) | Dilution (WB) | Dilution  (ICC) | Exposed time |
| --- | --- | --- | --- | --- | --- | --- |
| α-synuclein (C-20)-R | Santa Cruz | Rabbit | 1:500 | 1:500 |  | 4 hours |
| α-synuclein [LB 509] | Abcam | Mouse | 1:1,000 | 1:1,000 |  | 2 hours |
| α-synuclein (D37A6) | Cell Signaling Technology | Rabbit | 1:100 | 1:500 |  | overnight |
| α-synuclein (clone 4D6) | Signet | Mouse |  |  | 1:1,000  (o/n) | overnight |
| α-synuclein (phospho S129) | Abcam | Rabbit | 1:500 | 1:500 | 1:300 (o/n) | 4 hours |
| Glial fibrillary acidic protein (GFAP) | DakoCytomation | Rabbit |  |  | 1:800  (o/n) |  |
| Iba1 | Abcam | Goat |  |  | 1:2,000  (o/n) |  |
| Tyrosine Hydroxylase | Santa Cruz | Rabbit |  |  | 1:800 (o/n) |  |
| Monoclonal ANTI-FLAG® M2 | Sigma | Mouse | 1:1,000 | 1:10,000 | 1:300 (o/n) | 2 hours |
| Alexa 488 anti-mouse |  | Goat | 1:500 |  | 1:800  (o/n) | 45 min |
| Alexa 488 anti-rabbit |  | Goat | 1:500 |  | 1:2,000  (o/n) | 45 min |
| Alexa 594 anti-mouse |  | Goat | 1:500 |  | 1:800 (o/n) | 45 min |
| Alexa 594 anti-rabbit |  | Goat | 1:500 |  |  | 45 min |
